# Efficacy of single-and double-volume exchange transfusion for neonatal hyperbilirubinemia

**DOI:** 10.5937/jomb0-42668

**Published:** 2023-08-25

**Authors:** Zhongzheng Xiong, Xianchuan Liu, Xiangzhu Li

**Affiliations:** 1 Dian Jiang People's Hospital of Chongqing, Department of Medical Laboratory, Chongqing, China; 2 Bishan Maternity & Child Hospital of Chongqing, Laboratory Medicine, Chongqing, China; 3 Medical University, Bishan Hospital of Chongqing, Department of Blood Transfusion, Chongqing, China

**Keywords:** single-volume exchange transfusion, hyperbilirubinemia, newborn, efficacy, jednovolumenska transfuzija, hiperbilirubinemija, novorođenče, efikasnost

## Abstract

**Background:**

To investigate the efficacy and safety of singleand double-volume exchange transfusion for neonatal hyperbilirubinemia (HB) and compare their effects on the internal environment of newborns.

**Methods:**

The clinical data of 96 HB newborns admitted to and treated in our hospitals from January 2016 to October 2021 were retrospectively analyzed. Then, these newborns were divided into single volume group (80-110 mL/kg, n=48) and double volume group (150-180 mL/kg, n=48) by the exchange volume per unit body mass. The hematological indicators total serum bilirubin (TSB), peripheral blood red blood cell (RBC) count, white blood cell (WBC) count, platelet (PLT) count, serum albumin (ALB), prothrombin time (PT) and activated partial thromboplastin time (APTT), and changes in inner-environment indexes (blood gas, blood glucose, acid-base and electrolyte levels) were compared between the two groups of newborns before treatment and after once treatment. Additionally, the adverse reactions of exchange transfusion in the two groups of newborns were recorded.

## Abstract (sr)

**Uvod:**

Cilj je bio da se ispita efikasnost i bezbednost transfuzije izmene jednog i dvostrukog volumena za neonatalnu hiperbilirubinemiju (HB) i uporedi njihov efekat na unutrašnje okruženje novorođenčadi.

**Metode:**

Retrospektivno su analizirani klinički podaci 96 HB novorođenčadi primljenih i lećenih u našim bolnicama od januara 2016. do oktobra 2021. godine. Zatim su ova novorođenčad podeljena u jednovolumensku grupu (80-110 mL/kg, n=48) i grupu sa dvostrukim zapreminom (150-180 mL/kg, n=48) prema zapremini razmene po jedinici telesne mase. Hematološki indikatori ukupni bilirubin u serumu (TSB), broj crvenih krvnih zrnaca u perifernoj krvi (RBC), broj belih krvnih zrnaca (WBC), broj trombocita (PLT), serumski albumin (ALB), protrombinsko vreme (PT) i aktivirani parcijalni tromboplastin vreme (APTT), i upoređene su promene u indeksima unutrašnje sredine (gas u krvi, glukoza u krvi, kiselinsko-bazni i nivoi elektrolita) između dve grupe novorođenčadi pre tretmana i posle tretmana. Pored toga, zabeležene su i neželjene reakcije razmene transfuzije u dve grupe novorođenčadi.

**Rezultati:**

Srednja zapremina razmene bila je (96,79± 11,52) mL/kg i (160,74±10,19) mL/kg, a vreme razmene (98,66±19,86) min i (110,33±22,71) min u grupi sa jednom zapreminom i dvostrukom zapreminom grupa, respektivno. Razlike su bile statistički značajne (P=0,009). Prosečna dužina boravka u bolnici bila je (9,14±3,78) d u grupi sa jednom zapreminom i (9,75±4,05) d u grupi sa dvostrukim volumenom, ne pokazujući statistički značajnu razliku (P=0,448). Nivoi ukupnog bilirubina (TBIL) i indirektnog bilirubina značajno su opali nakon transfuzije razmene u poređenju sa onima pre razmene transfuzije (P=0,032), i bili su značajno niži u grupi sa dvostrukom zapreminom od onih u grupi sa jednom zapreminom nakon transfuzije (P=0,007). Kurs razmene TBIL-a bio je značajno viši u grupi sa dvostrukim volumenom nego u grupi sa jednim zapreminom (58,60±3,73)% naspram (50,57±3,45)%, P=0,023. U poređenju sa onima pre razmene transfuzije, broj belih krvnih zrnaca, broj PLT, snaga vodonika (pH), nivo HCO3, i nivoi natrijuma u serumu, kalijuma u serumu, serumskog kalcijuma i ALB su značajno smanjeni, dok su broj eritrocita, PT, APTT značajno smanjeni, a nivo glukoze u krvi je značajno povećan nakon transfuzije razmene. Nivo hemoglobina (Hb), PaO2 i PaCO2 u dve grupe posle razmene transfuzije nisu pokazali statistički značajne razlike u odnosu na one pre razmene (P>0,05). Nakon transfuzije, grupa sa dvostrukim volumenom pokazala je značajno smanjen broj PLT i značajno povišen nivo glukoze u krvi za razliku od grupe sa jednom zapreminom (P=0,019), a nije bilo statistički značajnih razlika u ostalim pokazateljima između dve grupe (P> 0,05). Neželjene reakcije povezane sa razmenom transfuzije uglavnom su uključivale hiperglikemiju, poremećaje acidobazne i elektrolitske vrednosti, apneju, nekrotizirajući enterokolitis (NEC) i srčanu insuficijenciju. Sva novorođenčad su poboljšana i otpuštena nakon simptomatske terapije. Nije pronađena statistički značajna razlika u stopi incidencije neželjenih reakcija između dve grupe (P>0,05).

**Zaključak:**

Za neonatalni HB, transfuzija izmene jedne zapremine ima manje uticaja na unutrašnju sredinu novorođenčadi, potrebna je manja zapremina potrošnje krvi i kraće vreme razmene i može vidljivo da snizi nivo bilirubina u serumu u poređenju sa transfuzijom izmene dvostruke zapremine. Dakle, jednovolumenska transfuzija ima povoljnu vrednost u kliničkoj primeni.

## Introduction

Neonatal jaundice refers to visible skin, mucous membrane and sclera stained yellow caused by increased serum bilirubin concentration, which has a relatively high incidence rate in the neonatal period [1]. Severe hyperbilirubinemia (HB) may lead to neonatal bilirubin encephalopathy and neurological sequelae. Early detection and timely intervention of severe HB can largely prevent the occurrence and development of brain injury [2]. Neonatal severe HB is commonly treated by phototherapy. In the case of toxicity of bilirubin to the central nervous system or obvious clinical signs of acute bilirubin encephalopathy (ABE) after phototherapy, arteriovenous synchronous exchange transfusion can quickly reduce the bilirubin in blood and relieve and avoid the toxicity of bilirubin to the central nervous system [3] [4] [5]. As to exchange transfusion, the exchange volume is an important factor affecting its efficacy. Based on anaylsis results, the exchange volume is positively correlated with the decrease in the bilirubin level after exchange transfusion. Besides, it is also reported that a large exchange volume is also a major factor for the high incidence rate of complications. Decreasing the exchange volume can not only achieve better therapeutic effects, but also reduce the incidence rate of complications [6] [7] [8].

In this paper, the clinical data of AML (non-APL) newborns admitted to and treated in our hospital were retrospectively analyzed, and the characteristics of the disease, effects of chemotherapy and risk fractors of prognosis were summarized, so as to provide a strong basis for the treatment of newborns.

## Materials and methods

### Study subjects

A total of 96 HB newborns admitted to and treated with exchange transfusion in the Neonatal Center of our hospital from January 2016 to October 2021 were enrolled. The inclusion criteria were set as follows: 1) Newborns with total serum bilirubin (TSB) meeting or exceeding the reference standard of exchange transfusion recommended by American Academy of Pediatrics in 2004 [9], 2) those with an age of 28 d and a gestational age of 35 weeks, 3) those with no significant effect after phototherapy, and 4) those receiving exchange transfusion for the first time. The following were exclusion criteria: 1) Newborns with obvious manifestations of bilirubin encephalopathy before exchange transfusion, 2) those with severe systemic infections that were not be effectively controlled, or 3) those with congenital malformations. Among the 96 newborns enrolled in this study, there were 39 males and 57 females, with a mean gestational age of (39.11±1.51) weeks, an average age of (4.42±1.77) days and an average body weight of (3.45±0.50) kg. All newborns enrolled were informed and signed the informed consent in accordance with Declaration of Helsinki. This study was approved by the Ethics Committee of our hospital.

### Therapeutic methods

Before exchange transfusion, the newborns were treated with phototherapy under new cold light source LEDs (wavelength: 450–470 nm, and light intensity: >30 W cm^-2^ nm^-1^) for 4–6 h. Then, ex - change transfusion was conducted immediately if the TSB level had no decrease or even a continuous increase or the decrease in the TSB level in newborns with immune hemolysis was less than 2–3 mg·dL^-1^.

The following two blood sources could be selected: mixed maternal RH homotypic blood of red blood cells (RBCs) and plasma at a ratio of 1:1 and recombinant blood (O RBCs + AB plasma). According to the results of hepatic function examination before exchange transfusion, whether to use human albumin (ALB) before exchange transfusion was determined. If the ALB level was less than 25 g·L-1, ALB (1 g·kg^-1^) was given to increase the connection between bilirubin and ALB and reduce the free bilirubin in blood. If the ALB level was normal, no additional ALB was needed. The newborns were placed on a rescue table under far-infrared radiation to monitor heart rate, respiration, oxygen saturation and blood pressure, routinely deprived of food once before surgery, and sedated with chloral hydrate. A gastric tube was indwelled, and peripheral arteriovenous channels were established. As for the exchange volume, single volume was set as 80–110 mL/kg, and double volume as 150–180 mL/kg for calculation. The manual peripheral arteriovenous double-tube synchronous exchange transfusion was adopted for all newborns at a rate of 3–5 mL/min. About 10 mL of blood was drawn from the arterial end before and after ex - change transfusion to detect indexes including bilirubin hepatic and renal functions, blood gas, blood glucose and electrolytes. During exchange transfusion, the changes in vital signs, complexion and blood oxygen saturation were closely monitored. After exchange transfusion, newborns were sent to the intensive care unit for such treatment as blue light phototherapy, blood alkalization and ALB infusion.

### Observation indexes

Before treatment and after once treatment, 10 mL of blood sample was collected from the arterial duct of newborns. Then, some whole blood was retained for immediate detection of RBC count, white blood cell (WBC) count, platelet (PLT) count, PT and APTT using a hematology analyzer and a semi-automatic coagulation analyzer. Some blood samples were fully coagulated at low temperature and centrifuged at high speed. Then, the upper-layer serum was collected, and the concentrations of total bilirubin (TBIL), indirect bilirubin and ALB therein were determined using corresponding kits through enzyme-linked immunosorbent assay (ELISA).

Next, the changes in the hematology and metabolism indicators [TBIL, RBC, WBC, PLT, hemoglobin (Hb), ALB, PT, APTT, power of hydrogen (pH), PaO_2_, PaCO_2_, HCO_3_^-^, serum sodium, serum potassium, serum calcium, blood glucose and serum ALB levels] were compared between the two groups of newborns before treatment and after once treatment. The first tube of blood drawn at the beginning of exchange transfusion and blood samples collected at the end of exchange transfusion were preserved for determination of the TSB. The bilirubin exchange rate = (bilirubin before exchange transfusion – bilirubin after exchange transfusion)/bilirubin before exchange transfusion ×100%.

Moreover, the incidence of exchange transfusion- related adverse reactions [including apnea or respiratory failure, acute renal failure, necrotizing enterocolitis (NEC), shock, disseminated intravascular coagulation (DIC), heart failure or cardiac arrest, and death] were recorded.

### Statistical analysis

Statistical Product and Service Solutions (SPSS) 22.0 (IBM, Armonk, NY, USA) was utilized for statistical analysis. Measurement data were expressed as mean ± standard deviation (FORMULAx±s), and t test was adoped for comparison between two groups. Enumeration data were expressed as ratio (%) and compared through 2 test or Fisher’s exact probability test. Measurement data not conforming to normal distribution were represented as the median and interquartile range [M (P25, P75)], the Mann-Whitney U test was used for comparison among groups, and the Wilcoxon rank sum test was applied for comparison within groups. P<0.05 indicated that the difference was statistically significant.

## Results

### Comparison of efficacy of exchange transfusion between the two groups of newborns

The mean exchange volume and the exchange time were (96.79±11.52) mL/kg and (98.66± 19.86) min, respectively, in single volume group and (160.74±10.19) mL/kg and (110.33±22.71) min, respectively, in double volume group, showing statistically significant differences (P=0.009). The average length of hospital stay was (9.14±3.78) d and (9.75±4.05) d in single volume group and double volume group, respectively, displaying no statistically significant difference (P=0.448) (Table 1). Before exchange transfusion, the TBIL and indirect bilirubin levels in serum displayed no statistically significant differences between the two groups (P>0.05). After exchange transfusion, they were significantly decreased compared with those before exchange transfusion (P=0.032), and they were significantly lower in double volume group than those in single volume group (P=0.007). The TBIL exchange rate was significantly higher in double volume group than that in single volume group, and the difference was statistically significant [(58.60±3.73)% vs. (50.57± 3.45)%, P=0.023]. The average time of phototherapy after exchange transfusion was (56.43±21.89) h in single volume group and (49.40±18.91) h in double volume group. The proportion of newborns receiving exchange transfusion again was 4.2% (2/48) and 2.1% (1/48) in single volume group and double volume group, respectively. The differences were not statistically significant (P=0.096, P=0.679) (Table 2).

**Table 1:** Baseline demographic and clinical characteristics of the studied children.

Parameters	Single volume group <br>n=48	Double volume group <br>n=48	P-value
Age (d)	4.17±1.87	4.68±1.69	0.164
Gestational age (week)	38.90±1.56	39.34±1.48	0.160
Gender (Male/Female)	22/26	17/31	0.406
Birth weight (kg)	3.39±0.54	3.54±0.47	0.150
Caesarean section (n, %)	14 (28.9%)	18 (28.9%)	0.516
Breast feeding (n, %)	28 (28.9%)	33 (28.9%)	0.397
Average exchange transfusion volume (mL/kg)	96.79±11.52	160.74±10.19	0.001
Exchange transfusion time (min)	98.66±19.86	110.33±22.71	0.009
Hospital stay time (d)	9.14±3.78	9.75±4.05	0.448

**Table 2:** Comparison of serum bilirubin levels before and after exchange transfusion of newborn in the two studied groups.

	Single volume group <br>n=48	Double volume group <br>n=48	P-value
Total serum bilirubin (mmol/L)			
Before exchange transfusion	421.21±60.75	434.27±57.16	0.281
After exchange transfusion	203.94±25.68	190.42±23.49	0.032
Indirect serum bilirubin (mmol/L)			
Before exchange transfusion	411.53±53.74	419.62±51.25	0.452
After exchange transfusion	193.57±26.63	178.85±25.44	0.007
Total serum bilirubin exchange rate	50.57%±3.45%	58.60%±3.73%	0.023

### Changes in blood indicators before and after exchange transfusion in the two groups

There were no statistically significant differences in peripheral blood RBC count, WBC count, PLT count, Hb level, PT and APTT between the two groups before exchange transfusion (P>0.05). Compared with those before exchange transfusion, the WBC count and PLT count were significantly reduced, while RBC count, PT and APTT were significantly increased after exchange transfusion, and the differences were of statistical significance (P<0.05). The Hb level in the two groups after exchange transfusion showed no statistically significant difference from that before exchange transfusion (P>0.05). After exchange transfusion, double volume group exhibited a significantly decreased PLT count in contrast with single volume group, and there were no statistically significant differences in the other indicators between the two groups (P>0.05), Table 3.

**Table 3:** Comparison of hematological indexes before and after exchange transfusion of newborns in the two studied groups. Notes: Hb: Hemoglobin; PT: Prothrombin time; APTT: Activated partial thromboplastin time.

	Single volume group <br>n=48	Double volume group <br>n=48	P-value
Red blood cells (×10^12^/L)			
Before exchange transfusion	4.02±0.95	4.26±0.86	0.198
After exchange transfusion	4.90±0.79	4.82±0.67	0.594
Hb (g/L)			
Before exchange transfusion	145.35±23.71	149.66±28.29	0.421
After exchange transfusion	153.16±36.43	151.88±35.51	0.567
White blood cells (×10^9^/L)			
Before exchange transfusion	14.74±6.03	14.04±4.84	0.397
After exchange transfusion	8.89±4.26	8.23±3.52	0.180
Platelets (×10^9^/L)			
Before exchange transfusion	296.49±66.33	307.73±79.05	0.114
After exchange transfusion	123.87±35.68	106.59±30.21	0.012
PT (M (P25, P75), s)			
Before exchange transfusion	14.71 (12.39, 16.06)	14.43 (12.76, 15.95)	0.588
After exchange transfusion	16.54 (14.70, 17.18)	16.46 (14.58, 17.55)	0.339
APTT (M (P25, P75), s)			
Before exchange transfusion	51.90 (42.37, 60.96)	50.89 (40.56, 62.31)	0.461
After exchange transfusion	105.29 (66.73, 130.77)	102.50 (63.45, 127.90)	0.127

### Changes in metabolism indicators before and after exchange transfusion in the two groups

Before exchange transfusion, the pH value, PaO_2_, PaCO_2_, HCO_3_^-^ level and serum sodium, serum potassium, serum calcium, blood glucose and serum ALB levels displayed no statistically significant differences between the two groups (P>0.05). Compared with those before exchange transfusion, the pH value, HCO_3_^-^ level and serum sodium, serum potassium, serum calcium and serum ALB levels significantly declined, whereas the blood glucose level significantly rose after exchange transfusion, and the differences were statistically significant (P<0.05). The PaO_2_ and PaCO_2_ levels in the two groups after exchange transfusion showed no statistically significant differences from those before exchange transfusion (P>0.05). After exchange transfusion, the blood glucose level was significantly higher in double volume group than that in single volume group (P=0.019), and no statistically significant differences were detected in the other indicators between the two groups (P>0.05), Table 4.

**Table 4:** Comparison of metabolic indexes before and after exchange transfusion of newborns in the two studied groups. Notes: pH: Pondus Hydrogenii; PaO_2_: Arterial partial pressure of oxygen; PaCO_2_: Arterial partial pressure of carbon dioxide.

	Single volume group <br>n=48	Double volume group <br>n=48	P-value
pH			
Before exchange transfusion	7.44±0.05	7.42±0.05	0.689
After exchange transfusion	7.33±0.07	7.35±0.06	0.690
PaO_2_ (mmHg)			
Before exchange transfusion	77.80±25.62	79.34±20.24	0.251
After exchange transfusion	82.22±20.48	85.65±35.41	0.177
PaCO_2_ (mmHg)			
Before exchange transfusion	34.04±6.63	36.69±6.94	0.363
After exchange transfusion	35.73±7.27	37.33±7.58	0.230
HCO_3_^-^ (×10^9^/L)			
Before exchange transfusion	22.49±3.83	21.70±4.01	0.413
After exchange transfusion	19.37±3.64	19.19±3.25	0.361
Serum Na (mmol/L)			
Before exchange transfusion	140.67±4.07	140.13±4.54	0.543
After exchange transfusion	132.25±5.15	133.73±4.11	0.232
Serum K (mmol/L)			
Before exchange transfusion	4.21±0.55	4.60±0.49	0.275
After exchange transfusion	4.10±0.65	4.31±0.63	0.491
Serum Ca (mmol/L)			
Before exchange transfusion	2.24±0.20	2.26±0.29	0.794
After exchange transfusion	2.02±0.30	2.07±0.31	0.544
Blood glucose (mmol/L)			
Before exchange transfusion	5.75±1.34	5.91±1.94	0.449
After exchange transfusion	8.85±1.76	9.40±2.18	0.019
Albumin (g/L)			
Before exchange transfusion	36.26±3.78	35.35±5.10	0.376
After exchange transfusion	24.64±4.96	22.86±5.41	0.121

### Incidence rate of adverse reactions in newborns

All exchange transfusion-related adverse reactions occurred after exchange transfusion. There were 1 case and 4 cases of hyperglycemia, 1 case and 2 cases of metabolic acidosis, 2 cases and 3 cases of hyperkalemia, 3 cases and 5 cases of hyponatremia, 2 cases and 3 cases of hypocalcemia, 1 case and 2 cases of hypokalemia, 3 cases and 4 cases of apnea, 0 case and 2 cases of NEC, and 0 case and 1 case of heart failure in single volume group and double volume group, respectively. All newborns had alleviated adverse reactions and were discharged after symptomatic therapy. No severe complications such as acute renal failure, shock, DIC, cardiac arrest and death were detected. There was no statistically significant difference in the incidence rate of adverse reactions between the two groups (P>0.05).

## Discussion

HB is one of the common diseases leading to the hospitalization of newborns, with an incidence rate of 50–70% in full-term newborns and 80% in preterm infants [10]. Due to the neurotoxicity of high level of TSB, severe neonatal HB without timely treatment will result in such complications as bilirubin encephalopathy, cerebral palsy, sensorineural hearing loss, ocular motility disorders and enamel dysplasia, and even death of newborns [11]. Currently, treatment methods for neonatal HB mainly include phototherapy, exchange transfusion and drug therapy [12]. Exchange transfusion for newborns has been widely applied in clinical practice since the 1970s when it was put forward. Single-channel umbilical venesection used for manual blood transfusion and drawing in the past leads to many adverse reactions of the gastrointestinal and blood systems. At present, it has been replaced by arteriovenous double-tube synchronous exchange transfusion that is implementated by simple puncture and control via pump injection devices to avoid making incisions and excessive fluctuation of blood volume [13]. Peripheral arteriovenous automatic exchange transfusion can quickly and effectively remove free bilirubin, sensitized RBCs and antibodies in blood, which is one of the effective approaches to the treatment of neonatal HB. The blood volume of newborns is usually about 80 mL·kg- 1, and double circulating blood volume exchange transfusion can exchange about 80–90% of sensitized RBCs and reduce 60% of circulating bilirubin and antibodies [14] [15]. Therefore, double circulating blood volume exchange transfusion is the first applied in clinic. However, it is found in clinical application that single circulating blood volume exchange transfusion has a lower bilirubin exchange rate than double circulating blood volume exchange transfusion, but its advantages are very obvious, namely, it is capable of reducing the exchange volume, maintaining the stability of the internal environment, decreasing the adverse reactions of blood transfusion, and saving blood.

In this study, the bilirubin exchange rate was (41.68±8.52)% and (50.22±13.14)% in single volume group and double volume group, respectively, and the reduction in bilirubin was larger in double volume group, suggesting that the reduction in bilirubin may have a positive association with the exchange volume, but they are not proportionally elevated. The decline in bilirubin was also obvious in single volume group, and better therapeutic effect was achieved. In addition, the internal environment changed to a certain extent after both single- and double-volume exchange transfusion, mainly manifested as reductions in WBC and PLT, hypoproteinemia, hypokalemia, hyponatremia, hypochloremia, and hypocalcemia, hyperglycemia and metabolic acidosis, consistent with the findings reported in relevant literature [16]. However, the changes in PLT and blood glucose in single volume group were smaller than those in double volume group, implying that the changes in these two indicators are greatly affected by the exchange volume. The possible reason is that mixed blood of RBC suspension and plasma is mainly used for exchange transfusion, in which the PLT concentration is very low. Currently, the maintenance solution of RBC suspension is mainly dextrose citrate, and the blood glucose concentration is (27.10± 2.01) mmol/L on the day of storage and (16.614- 3.99) mmol/L at 35 d after storage, which is much higher than the physiological level of human blood glucose. The larger exchange volume has a greater impact on the blood glucose level of body. Most of the changes in the internal environment are temporary and reversible, but they are a major stress for high-risk newborns or premature infants. Besides, it is difficult to relieve the internal environmental imbalance caused by exchange transfusion through self-regulation in a short time. As a result, irreversible damage and even death occur [17] [18]. Therefore, it is meaningful to improve the effect of exchange transfusion on the internal environment by reducing the exchange volume.

In addition, the resutls of this study showed that there were more adverse reactions in double volume group than single volume group, but the difference was not statistically significant (P>0.05). The hemodynamic changes caused by exchange transfusion, especially the large fluctuation of blood pressure during exchange transfusion, can easily lead to ischemic damage to the nervous system, which may be one of the factors leading to apnea after exchange transfusion [19]. If newborns have congenital heart disease and weakened pump function of the heart, the instability of the systemic circulation during exchange transfusion will increase the cardiac load, which may lead to heart failure. In this study, heart failure was detected in only 1 newborn who was discharged after rescue. The hemodynamics of newborns is extremely unstable during exchange transfusion, and the increased number of times of exchange transfusion will induce the superimposed effect of the instable circulatory system. Therefore, it is necessary to carefully evaluate the indications for exchange transfusion and crucial to monitor the vital signs of newborns during exchange transfusion. Besides, the exchange transfusion shall be slowed down or even terminated if necessary. During exchange transfusion, some blood in the blood circulation may be redistributed, causing insufficient intestinal perfusion and thus leading to NEC and stress ulcers. Hence, after exchange transfusion, the colour of stool and signs of the abdomen shall be closely observed, and abdominal imaging examination shall be carried out if necessary [20] [21].

This study is a retrospective study with limited sample size and incomprehensive follow-up period and content. Hence, multicenter and large-sample prospective randomized studies are needed in the future to verify the conclusion made in this study.

## Conclusions

For neonatal HB, single-volume exchange transfusion has fewer effects on the internal environment of newborns, needs smaller blood consumption volume and shorter exchange time and can visibly lower the serum bilirubin level in contrast with double-volume exchange transfusion. Hence, single-volume exchange transfusion has favorable value in clinical application.

## Dodatak

### Author contribution statement

Zhongzheng Xiong and Xianchuan Liu contributed equally to this work.

### Conflict of interest statement

All the authors declare that they have no conflict of interest in this work.
